# Factors affecting return to work after injury or illness: best evidence synthesis of systematic reviews

**DOI:** 10.1186/s12998-016-0113-z

**Published:** 2016-09-08

**Authors:** Carol Cancelliere, James Donovan, Mette Jensen Stochkendahl, Melissa Biscardi, Carlo Ammendolia, Corrie Myburgh, J. David Cassidy

**Affiliations:** 1 Institute of Health Policy, Management and Evaluation, Dalla Lana School of Public Health, University of Toronto, Toronto, Ontario Canada; 2 Institute of Health Policy, Management and Evaluation, Dalla Lana School of Public Health, University of Toronto, Toronto, Ontario Canada; 3 Nordic Institute of Chiropractic and Clinical Biomechanics, Campusvej 55, 5230 Odense M, Denmark; 4 Sports and Exercise Medicine Institute, Toronto, Ontario Canada; 5 Department of Surgery, Faculty of Medicine, University of Toronto, Toronto, Ontario Canada; 6 Department of Sports Science and Clinical Biomechanics, University of Southern Denmark, Campusvej 55, 5230 Odense M, Denmark; 7 Division of Epidemiology, Dalla Lana School of Public Health, University of Toronto, Toronto, Ontario Canada

**Keywords:** Return to work, Work disability, Prognosis, Intervention, Sick leave, Absenteeism, Presenteeism

## Abstract

**Background:**

Work disability is a major personal, financial and public health burden. Predicting future work success is a major focus of research.

**Objectives:**

To identify common prognostic factors for return-to-work across different health and injury conditions and to describe their association with return-to-work outcomes.

**Methods:**

Medline, Embase, PsychINFO, Cinahl, and Cochrane Database of Systematic Reviews and the grey literature were searched from January 1, 2004 to September 1, 2013. Systematic reviews addressing return-to-work in various conditions and injuries were selected. Eligible studies were critically appraised using the Scottish Intercollegiate Guidelines Network criteria to identify low risk of bias reviews.

**Results:**

Of the 36,193 titles screened and the 94 eligible studies reviewed, 56 systematic reviews were accepted as low risk of bias. Over half of these focused on musculoskeletal disorders, which were primarily spine related (e.g., neck and low back pain). The other half of studies assessed workers with mental health or cardiovascular conditions, stroke, cancer, multiple sclerosis or other non-specified health conditions. Many factors have been assessed, but few consistently across conditions. Common factors associated with positive return-to-work outcomes were higher education and socioeconomic status, higher self-efficacy and optimistic expectations for recovery and return-to-work, lower severity of the injury/illness, return-to-work coordination, and multidisciplinary interventions that include the workplace and stakeholders. Common factors associated with negative return-to-work outcomes were older age, being female, higher pain or disability, depression, higher physical work demands, previous sick leave and unemployment, and activity limitations.

**Conclusions:**

Expectations of recovery and return-to-work, pain and disability levels, depression, workplace factors, and access to multidisciplinary resources are important modifiable factors in progressing return-to-work across health and injury conditions. Employers, healthcare providers and other stakeholders can use this information to facilitate return-to-work for injured/ill workers regardless of the specific injury or illness. Future studies should investigate novel interventions, and other factors that may be common across health conditions.

## Introduction

Work disability is a major personal, financial and public health burden [1]. Annual productivity losses from missed workdays due to low back pain (LBP) are estimated at $28 billion in the United States alone [2] and LBP is now the leading cause of disability, affecting nearly 600 million people worldwide [3]. In addition to musculoskeletal disorders (MSKDs) [3, 4], disability from cardiovascular disease [5], cancer survivorship [6] and mental health disorders [7] are also increasing. With more people living with many types of chronic illnesses now than ever before, the problem of work disability will continue to escalate if we do not take action [8]. Return to work (RTW) is a major indicator of real-world functioning; thus, predicting future work success is a major focus of research [9]. Individuals unable to RTW due to an injury or illness can experience greater physical ailments, as well as poorer psychosocial adjustment (i.e., increased anxiety, depression, social isolation). [10, 11] The consequences studied in those exposed to sick leave include inactivity and isolation, suicide, decreased career opportunities, and decreased personal finances [12]. The consequences studied in those exposed to disability pension include reduced quality of life and self-reported health, and increased health service utilisation and substance abuse [12]. Improving return to work (RTW) outcomes is therefore critical.

Most of the RTW literature has focussed on MSKDs, especially LBP. “Seven principles for successful RTW” have previously been established for MSKDs by the Institute of Work and Health in 2007 [13]: (1) the workplace has a strong commitment to health and safety; (2) work accommodation; (3) support the returning worker without disadvantaging co-workers and supervisors; (4) supervisors are trained in work disability prevention and included in RTW planning; (5) the employer makes early and considerate contact with injured/ill workers; (6) RTW coordination; and (7) employers and healthcare providers communicate with each other about the workplace demands. These guidelines were intended for all workplaces and RTW professionals. Additionally, Briand et al. [14] synthesized systematic reviews of RTW interventions for MSKDs up to 2006. They indicated that the essential components of RTW interventions are centralized coordination of the worker’s RTW, formal individual psychological and occupational interventions, workplace based interventions, work accommodations, and contact between the various stakeholders and interventions. Many of these factors are closely related to the environment and workplace, and it is likely that these prognostic factors are not unique to MSKDs. As such, rather than investigating RTW prognostic factors for a multitude of different health conditions, there is a need to understand the common factors that determine RTW across these conditions. Often times, the specific disease-related or biomedical determinants are not the main drivers of patient-centred outcomes such as quality of life and employment. Moreover, identifying modifiable factors is important because certain socio-demographic (e.g., blue-collar work, older age) and some disease-related factors (e.g., presence of rheumatoid factor) fail to provide information on modifiable targets for RTW interventions even though they are important to identify those at risk for prolonged work disability.

Returning employees to work is complex and involves the interplay of many factors beyond disease. We do not know, however, if any of these factors are common across conditions and might form the basis for generic RTW strategies that can be tested and broadly applied across conditions and settings, or even applied to more rare conditions that have not been studied. To the best of our knowledge no systematic review has focused on this issue. The purpose of this systematic review is to identify and collate systematic reviews across health conditions and injuries to identify common prognostic factors of RTW and to describe their associations with RTW outcomes. This information has important implications for workers, healthcare professionals, work-disability professionals, supervisors, employers and insurers.

## Methods

We conducted a systematic review of systematic reviews and best evidence synthesis [15]. Our review was conducted in compliance with the Preferred Reporting Items for Systematic Reviews and Meta-Analyses (PRISMA) statement [16]. Our protocol was registered in PROSPERO (International Prospective Register of Systematic Reviews) [17] on December 5, 2012. The registration number is CRD42012003396 (http://www.crd.york.ac.uk/prospero/).

### Search and retrieval of systematic reviews

With the aid of an information specialist, a search strategy was produced for the peer-reviewed scientific literature in the electronic databases Medline, Embase, PsychINFO, Cinahl, and Cochrane Database of Systematic Reviews from 2004 to 2012 and was updated on September 1, 2013. We began the search in 2004 because we aimed to capture the most recent studies. The reference lists of eligible articles were reviewed to identify additional sources. We also systematically searched relevant databases (TRIP Database, ClinicalKey, OT seeker, PEDro, NICE, SIGN, and NHS) and the grey literature which included government and research websites.

### Eligibility criteria

All systematic reviews were screened for eligibility according to pre-defined criteria. Inclusion criteria were: 1) language: English); 2) publication type and study design: published, peer-reviewed systematic reviews (with or without meta-analyses) of quantitative primary studies (e.g., randomized controlled trials (RCTs), and cohort studies). If quantitative and qualitative studies were reported together, they were included if the quantitative and qualitative results were synthesized separately; 3) study population: working age or ≥ 18 years of age; 4) case definition: any work or non-work-related injury or illness; (5) prognostic factor: any measurement associated with RTW including interventions. Interventions can be of any type (e.g., clinical and disease-specific, generic, or multidisciplinary) as long as the outcome was related to RTW; and 6) study outcomes: RTW outcomes, e.g., delayed or quicker RTW, duration of sick leave, duration of work disability, or number of sickness absence events. Exclusion criteria were: 1) publication type: narrative reviews, letters, editorials, commentary, dissertations, books and book chapters, conference proceedings, meeting abstracts, lectures and addresses; and 2) study designs: primary studies, non-systematic reviews, systematic reviews of qualitative studies.

### Screening

For the first level of screening, one reviewer read the titles of all the citations retrieved from the electronic databases, grey literature, and reference list searches and removed all those citations not related to RTW. In the second level of screening, abstracts of the relevant citations were reviewed by one reviewer. Full text articles were obtained for all abstracts except for those that did not meet the eligibility criteria. If after analyzing the full text, the eligibility of an article was still uncertain, a second reviewer independently undertook a full-text analysis of the article to determine eligibility. A third reviewer was to be consulted in the event of any disagreements, but this was not required.

### Assessment of risk of bias

The internal validity of eligible systematic reviews was critically assessed by random pairs of independent reviewers using the Scottish Intercollegiate Guidelines Network (SIGN) criteria [18]. The domains we assessed included the search strategy, methods of study selection and data extraction, the assessment of scientific quality, and the method of data synthesis. For example, a systematic review was considered to have a low risk of bias if the research question was clearly defined, the inclusion/exclusion criteria were listed, the search strategy was comprehensive, two reviewers independently selected studies and extracted data, the scientific quality of the included studies was assessed and reported, and appropriate methods were used to combine the individual study findings. A consensus method was used to solve any disagreements about the risk of bias assessment. A third reviewer was to be consulted if disagreements persisted, however this was not required. Systematic reviews with adequate internal validity (i.e., a low risk of bias) were accepted and included in our evidence synthesis.

### Data extraction

Data from accepted papers were extracted and entered into evidence tables by two reviewers independently (Table 1). Final entries were done by consensus and a third reviewer was to be consulted if there was any disagreement, however this was not required. The data extracted included: (1) first author and year; (2) search date, databases, language and limiters; (3) population; (4); prognostic factors; and (5) study authors’ main conclusions. Some reviews reported on other outcomes in addition to RTW outcomes (e.g., clinical outcomes and quality of life), however, we only reported the conclusions regarding RTW outcomes.

**Table 1:** Characteristics of accepted systematic reviews

First author, year	Search date, databases, language, limiters	Population	Prognostic factors	Outcomes	Study authors’ main conclusions
Musculoskeletal Conditions					
Brox 2008	Up to 2006; MEDLINE; English; RCTs	Non-specific chronic LBP (>12 weeks)	Back schools (5 RCTs), brief education in the clinical setting (4 RCTs), fear-avoidance training (3 RCTs)	RTW/sick leave	Based on 12 RCTs. RTW: Brief education vs. usual care in the clinical setting: strong evidence. Back schools: moderate evidence that they are not more effective than no intervention, waiting list, usual care, or a cognitive-behavioural-based back school; conflicting evidence for back schools vs. no intervention. Fear-avoidance training: moderate evidence for effectiveness compared to usual care. Sick leave: Fear-avoidance training incorporated in rehabilitation programs consisting of cognitive intervention and exercises is not different form spinal fusion.
Campbell 2013	Up to November 2011; MEDLINE, EMBASE, PsycINFO, CINAHL, IBSS, AMED, BNI; English; prospective, case-control	Nonspecific LBP	Employment social support type (e.g., co-worker, supervisor, general support)	RTW	Based on 32 articles. Weak effects of employment support; greater levels of co-worker support and general work support were associated with less time to RTW.
Carroll 2010	1990-2010; MEDLINE, Allied and Complementary Medicine, Applied social Sciences Index and Abstracts, British Nursing Index, Business Source Premier, the Cochrane Library, Cinahl, Current Contents, International Bibliography of the Social Sciences, PsycINFO, Sociological Abstracts, Science and the Social science Citation Index, Health Economics Evaluation Database, NHS Economics Evaluation Database, EconLit, Web of Science; English RCTs, controlled intervention studies	Back pain	Interventions involving the workplace	RTW	Based on 8 RCTs and 1 non-randomized controlled trial. Moderate evidence that stakeholder participation (i.e., the employee, the workplace, occupational health professionals) and work modification are more effective than other workplace-linked interventions, including exercise. Early intervention was effective.
Clay 2010 [49]	1985-May 2009; MEDLINE, EMBASE, PsycINFO, CINAHL, AMED; English; prospective and retrospective cohort studies	Acute orthopaedic trauma	Sociodemographic factors, injury and treatment related factors, psychosocial factors, work-related factors	RTW, duration of work disability	Based on 15 studies. RTW: limited evidence for any factor. Duration of work disability: strong evidence for level of education and blue collar work; moderate evidence for self-efficacy, injury severity and compensation
Dick 2011	Up to 2008; MEDLINE, EMBASE, CINAHL, AMED, PEDro, Cochrane Library; English; RCTs, cohort studies, systematic reviews	Upper limb disorders (carpal tunnel syndrome, non-specific arm pain, extensor tenosynovitis, lateral epicondylitis)	Workplace intervention	Employment outcomes	Based on 4 studies (3 RCTs, 1 cohort study). Non-specific arm pain: limited evidence that multidisciplinary rehabilitation for was beneficial for workers absent at least 4 weeks.
Franche 2005 [26]	January 1990-December 2003; MEDLINE, EMBASE, CINAHL, PsycINFO, Sociological Abstracts, ASSIA (Applied Social Sciences Index and Abstracts), ABI (American Business Index); English, French; quantitative studies	MSK and other pain-related conditions	Workplace-based RTW intervention components: early contact with the worker by the workplace, work accommodation offer, contact between healthcare provider and the workplace, ergonomic work site visits, supernumerary replacements, RTW coordination	Work disability duration	Based on 10 studies (4 RCTs, 1 non-RCT, 3 cohort, 1 pre-post, 1 cross-sectional). Overall moderate-strong evidence that workplace-based RTW interventions can reduce work disability duration. Intervention components: strong evidence for work accommodation, and contact between healthcare provider and workplace; moderate evidence for early contact with worker by workplace (within first 3 months of onset of work disability), ergonomic work site visits, and presence of a RTW coordinator. Insufficient evidence to support the effect of supernumerary replacements and the sustainability of effects beyond 1 year.
Hansson 2004	Up to October 2002; Medline, PsycINFO, SSCI; English, Danish, Norwegian, Swedish; longitudinal studies	Back and neck disorders	Demographic, medical, physical workplace, psychosocial workplace, socioeconomic, work organization, factors	Sickness absence, RTW	Based on 48 studies. Factors with consistent, but limited, support: (a) heavy physical workload, bent or twisted working position, and low work satisfaction increases the risk for short- and long-term sick leave; (b) specific back diagnoses and previous sick leave due to back disorders increases the risk for short-and long-term sick leave; (c) female gender, smoking, exposure to vibration, and deficient social support were not found to increase the risk for short- and long-term sick leave; (d) self-reported pain and functional impairments were associated with a high risk for long-term sick leave; (e) longer employment periods reduced the risk for short-term sick leave; (f) perceived demands at work did not influence short-term sick leave; (g) female gender and higher age increases the risk for disability pension.
Heymans 2005	Up to November 2004; MEDLINE, EMBASE, Cochrane Central Register of Controlled Trials; English, Dutch, French, German; RCTs	Non-specific LBP	Back schools	RTW	Based on 4 RCTs. Moderate evidence that back schools in an occupational setting improve RTW in the short- and intermediate-term, compared with exercises, manipulation, myofascial therapy, advice, placebo, or waiting list controls, for patients with chronic and recurrent LBP.
Hlobil 2005	Up to February 2004; MEDLINE, PsycINFO, EMBASE, Cochrane Controlled Trials Register; English; RCTs	Subacute, nonspecific LBP (with or without referral to the leg)	Out-patient interventions aimed at RTW (e.g., physical exercise or advice about it and education, behavioral treatment, ergonomic measures, case management).	RTW rate, days of work absenteeism	Based on 9 RCTs. Strong evidence for RTW interventions on the RTW rate after 6 months, and on the reduction of days of absence from work after ≥ 12 months. Conflicting evidence for the RTW rate after ≥ 12 months and on days of work absenteeism at 6 months.
Hoffman 2007	Up to October 2004; MEDLINE, PsycINFO, EMBASE, CENTRAL, CINAHL; English; RCTs	Noncancerous chronic LBP (≤3 months)	Psychological interventions	Employment/ disability compensation status	Based on 5 RCTs. Moderate evidence that multidisciplinary approaches that included a psychological component, when compared with active control conditions, have positive long-term effects on RTW (effect size 0.53, p<.05).
Iles 2008 [31]	Up to April 2006; MEDLINE, EMBASE, PsycINFO, CINAHL, PEDro; English; retrospective studies were excluded	Non-specific LBP (≤3 months)	Psychosocial variables	RTW	Based on 24 studies. Strong evidence that recovery expectation predicts work outcome and that depression, job satisfaction and stress/psychological strain do not predict work outcome. Moderate evidence that fear avoidance beliefs predict work outcome, and that anxiety does not predict work outcome. Insufficient evidence to determine whether compensation or locus of control predict work outcome.
Kent 2008	Up to 2007; MEDLINE, CINAHL, EMBASE, PsycINFO, AMED; English; prospective cohort studies	Non-specific LBP (<12 weeks)	Varying prognostic factors and interventions (psychosocial, history, pain, physical impairment, activity limitation, participation restriction, clinician factors)	Duration of compensation, time-off-work, return-to-full-work duties, RTW duties, time-off work.	Based on 50 studies. Conflicting and incomplete findings.
Kuijer 2006 [30]	Up to October 2004; MEDLINE, EMBASE, CINAHL, AMED, PsycINFO, Cochrane; Dutch, English, German; cohort studies, RCTs	Non-specific chronic LBP	Factors: sociodemographic, lifestyle, medical history, pain, observed disability, self-reported disability, health beliefs, physical work demands, psychological work demands, emotions, expectations.	Decision to report sickness absence or RTW	Based on 4 cohort studies and 13 RCTs. Consistent (strong) evidence for own expectations of recovery predicted decision to RTW. Patients with higher expectations had less sickness absence. No core set of predictors exists for sickness absence in general.
Kuijpers 2004	Up to February 2003; MEDLINE, EMBASE, CINAHL, PsycINFO, Sportdiscus; English; cohort studies	Shoulder complaints	Factors: worker group (blue vs. white collar), previous sick leave, duration of symptoms, continuous high intensity pain, pain with head rotation, pain with arm abduction	Sick leave	Based on 6 studies. Inconclusive.
Liddle 2007	1985-September 2004; MEDLINE, AMED, CINAHL, PsycINFO, Cochrane-Dare and Central Register of Controlled Trials, PubMed; RCTs	LBP	Use of advice in the management of LBP (e.g., to promote an understanding of LBP, and the importance of the patient playing an active role in their recovery).	Work disability	Based on 14 RCTs. Strong-medium support that advice as an adjunct to exercise is most effective for improving work disability in chronic LBP but, for acute LBP, is no more effective for improving work disability than simple advice to stay active.
Macedo 2010	Up to February 2009; MEDLINE, CINAHL, PsychINFO, PEDro, EMBASE; no language restriction; RCTs or quasi-randomized controlled trials	Non-specific LBP(persistent >6 weeks or recurrent)	Graded activity or graded exposure	RTW	Based on 3 RCTs. Conflicting evidence that graded activity vs. a minimal intervention provides faster RTW.
Meijer 2005	January 1990-December 2004; MEDLINE, EMBASE, PsycINFO, CINAHL; English; RCTs, clinical controlled trials, systematic reviews	Non-specific MSK complaints (mostly LBP)	RTW treatment programs: (1) knowledge conditioning (e.g., education, information); (2) physical conditioning (e.g., fitness exercises, graded activity exercise training); (3) psychological conditioning (e.g., cognitive behavioral techniques, coping skills); (4) social conditioning (training of social skills); (5) work conditioning (e.g., vocational training, workplace- based interventions)	RTW	Based on 18 studies (22 treatment programs). Inconsistent evidence. Seven experimental treatments resulted in faster RTW compared to control. Effective components: knowledge, psychological, physical and work conditioning, possibly supplemented with relaxation exercises. No negative findings .
Norlund 2009	April 1998-December 2006; PubMed; English; RCTs, controlled clinical trials	LBP: subacute (5-11 weeks) or chronic (≥12 weeks)	Multidisciplinary interventions	RTW	Based on 5 studies. Strong evidence that multidisciplinary interventions have a significant effect on RTW (RR 1.21, 95% CI 1.13-1.31).
Oesch 2010	Up to August 2008; MEDLINE, EMBASE, PEDro, Cochrane Library databases, NIOSHTIC-2, PsycINFO; language restrictions not specified; RCTs	Non-acute non-specific LBP (≥4 weeks)	Exercise (alone or as a part of multidisciplinary treatment)	Work disability	Based on 23 RCTs. Strong evidence in favour of exercise on work disability in the long term (OR 0.66, 95% CI 0.48-0.92) but not in the short and intermediate term. No conclusions regarding exercise types.
Palmer 2012	1990- April 2010; MEDLINE, EMBASE; RCTs, cohort studies	MSK disorders	Community- and workplace-based interventions: exercise therapy, behavioural change techniques, workplace adaptations, provision of additional services	RTW (27 studies), sickness absence (21), job loss (5)	Based on 42 studies (34 RCTs). Limited evidence that most interventions were beneficial (benefits are small): median RR for RTW was 1.21 (IQR 1.00-1.60), median RR for avoiding job loss was 1.25 (IQR 1.06-1.71), median RR for reduction in sickness absence was 1.11 (IQR 0.32-3.20) days/month. No intervention was clearly superior; effort-intensive interventions were less effective than simple ones.
Ravenek 2010	July 1998-July 2009; PubMed, EMBASE, SCOPUS, CINAHL, PsycINFO, Cochrane Library; English; RCTs, controlled clinical trials	Chronic LBP (≥12 weeks)	Multidisciplinary programs	Employment outcomes	Based on 12 trials. Conflicting evidence. Occupational therapists were underutilized.
Schaafsma 2013	Up to April 2012; CENTRAL, MEDLINE, EMBASE, CINAHL, PsycINFO, PEDro; CBRG Trials Register, ClilnicalTrials.gov, World Health Organization (WHO) International Clinical Trials Registry Platform (ICTRP); no language restrictions; RCTs	Back pain: acute (<6 weeks), subacute (6-12 weeks), or chronic (>12 weeks)	Physical conditioning as part of a RTW strategy	Work status outcomes	Based on 41 articles reporting on 25 RCTs. Low-moderate evidence: Light physical conditioning has no effect on sickness absence duration for workers with subacute or chronic back pain. Conflicting results for intense physical conditioning for workers with subacute back pain. Intense physical conditioning probably had a small effect on reducing sick leave at 12 months follow-up compared to usual care for workers with chronic back pain. Involving the workplace, or physical conditioning being part of integrated care management may have had a positive effect on reducing sick leave.
Steenstra 2005 [47]	1966 – December 2003; MEDLINE; no language restrictions specified; inception cohort studies	Acute LBP	Factors related to: pain, worker and workers’ health, psychosocial in worker and work, work organization, policy	Duration of sick leave	Based on 18 publications (14 cohorts). Moderate-strong evidence: specific LBP, higher disability levels, older age, female gender, more social dysfunction and more social isolation, heavier work, and receiving higher compensation predicted a longer duration of sick leave. A history of LBP, job satisfaction, educational level, marital status, number of dependants, smoking, working more than 8 hour shifts, occupation, and size of industry or company did not influence duration of sick leave due to LBP.
Tveito 2004 [25]	1980-November 2002; MEDLINE Advanced, PsycINFO, ISI base, Cochrane Controlled Trials Register; English; controlled trials	Employees (no further description)	LBP interventions at the workplace: preventive (educational, exercise, back belts, multidisciplinary, pamphlet), treatment	Sick leave	Based on 31 publications from 28 interventions (24 preventive, 4 treatment). Limited evidence: exercise interventions effect sick leave; multidisciplinary interventions have no effect. Moderate evidence:comprehensive treatment interventions No evidence for effect on sick leave: educational interventions, back belts, pamphlet.
van der Giessen 2012	Up to July 2011; PubMed, EMBASE, CINAHL, the Cochrane Library; no language restrictions; RCTs	Non-specific LBP	Graded activity	RTW	Based on 5 RCTs. Conflicting evidence that graded activity results in better RTW.
van Geen 2007 [23]	Up to April 2003; MEDLINE, Embase, Cochrane Controlled Trial register, PubMed, Psychlit; language restrictions not specificied; RCTs	Chronic non-specific LBP (≥12 weeks)	Multidisciplinary back training (including one physical and at least one other component: psychological, behavioral, educational or social)	Work participation (ability to work, number of days of sick leave, RTW)	Based on 5 studies. Strong evidence for positive long-term effect. Moderate evidence that the intensity of the intervention does not influence its effectiveness.
van Middelkoop 2011	Up to December 2008; MEDLINE, EMBASE, CINAHL, CENTRAL, and PEDro; English, Dutch, German; RCTs	Chronic non-specific LBP (≥12 weeks)	Physical and rehabilitation interventions	RTW, sick leave	Based on 3 studies. Low to moderate quality evidence that behavioural therapy and multidisciplinary treatment reduces sick leave.
Verkerk 2012	Up to March 2010; PubMed, CINAHL, EMBASE; RCTs, randomized cohort designs	Chronic non-specific LBP (≥12 weeks)	Factors: personal, health, pain, social, work, physical, psychological	RTW	Based on 8 studies. At baseline, there was limited evidence of a positive influence of lower pain intensity and physical job demands on RTW. At long-term follow-up, there is conflicting evidence for the association between RTW and age, sex, and activities of daily living.
Williams 2007	1982-April 2005; MEDLINE, CINAHL, EMBASE, AMED; English; prospective or cross-sectional designs	MSK LBP injuries	Workplace rehabilitation interventions involving secondary prevention	RTW	Based on 15 articles (10 studies). Limited evidence: clinical interventions with occupational interventions, and early RTW/modified work interventions were effective. These studies included early contact with the worker by the workplace and a health care provider intervention at the workplace. Ergonomic interventions (participatory ergonomics, workplace adaptation, adaptation of job tasks , adaptation of working hours) are effective.
Musculoskeletal and Other Conditions					
Corbière 2006	1985-2005; Cochrance Central Register of Controlled Trials, Cochrance Database of Systematic Reviews, MEDLINE, EMBASE, CINAHL, PsycINFO; English, French	Mental health problems and/or physical injuries (mostly MSK)	Psychological RTW interventions (e.g., cognitive behavioural therapy, communication skills)	Work outcomes	Based on 14 studies (4 RCTs, 2 controlled trials, 5 trials without randomization or control group, 1 evaluation only, 1 case study). Moderate-strong evidence of significant improvement in RTW.
Désiron 2011	1980-September 2010; CINAHL, Cochrane Library, EBSCO, MEDLINE (PubMed), PsycINFO; English; RCTs, cohort studies	Patients of working age that had participated in a rehabilitation program	RTW multidisciplinary rehabilitation programs that included occupational therapy (i.e., the therapeutic efforts had to be part of a defined program whose specific goal was to help patients re-enter or remain in the work force)	Work-related outcomes, e.g., RTW, sick leave, or employment status	Based on 3 RCTs and 3 cohort studies. Sufficient evidence that intervention contributes to RTW. Not clear what the effective components of the intervention are, except for workplace interventions.
Gensby 2012	Up to July 2010; MEDLINE, EMBASE, CINAHL, The Cochrane Library, SocINDEX, Social Services Abstracts, Sociological Abstracts, PsycINFO, EconLit, Business Source Elite, Safety Science and Risk, Dissertation Abstracts International (DAI); no language restrictions; RCTs, quasi experimental designs, single group designs	Employees on sick leave with injuries or illnesses (occupational or non-occupational)	Workplace disability management programs	RTW	Based on 13 studies (2 non-randomized studies, 11 single group ‘before and after’ studies). MSK disorders: 10, mental health conditions: 2. Lack of evidence on the effectiveness of programs.
Hoefsmit 2012	1994-2019; PubMed, CINAHL, Cochrane Library, Google Scholar; English; empirical studies or systematic literature reviews	Multiple groups, e.g., physical complaints, psychological complaints	RTW interventions	RTW	Based on 18 quantitative studies and 5 systematic reviews. *Early* interventions (initiated in first 6 weeks of sickness absence) are effective in multiple groups. *Multidisciplinary* interventions: effective in physical and psychological complaints. T*ime-contingent* interventions: effective in physical complaints; inconsistent evidence for psychological complaints. *Activating* interventions: effective in physical complaints (not studied for other complaints). Inconsistent evidence: targeting at employees with specific diagnoses, interventions of varying intensity and interventions covering employee and/or employer decision latitude. No positive effect: generic interventions targeted at all employees on sick leave.
Schandelmaier 2012 [46]	Up to April 2012; MEDLINE, EMBASE, CINAHL, PsycINFO, Cochrane Central Register of Controlled Trials; limiters not described; RCTs	Employees on work absence for at least 4 weeks.	RTW coordination programs	RTW	Based on 9 RCTs (8 MSK complaints, 1 mental health complaint). Moderate evidence: improves proportion at work at end of follow-up (risk ratio = 1.08, 95% CI = 1.03-1.13; absolute effect = 5 in 100 additional individuals returning to work, 95% CI = 2-8).
van Oostrom 2009	Up to November 2007; Cochrane Occupational Health Field Trials Register, CENTRAL, MEDLINE, EMBASE, PsycINFO; RCTs; no restrictions by date, language or publication status	MSK disorders, mental health problems, and other health conditions	Workplace interventions focusing on changes in the workplace or equipment, work design and organization, working conditions or environment, and occupational (case) management with active stakeholder involvement of the worker and the employer.	Sickness absence	Based on 6 RCTs: MSK disorders (5), mental health problems (1). Moderate evidence supports the use of workplace interventions to reduce sickness absence among workers with MSK disorders when compared to usual care. Not possible to investigate the effectiveness of workplace interventions among workers with mental health problems and other health conditions due to a lack of studies.
Mental Health Conditions					
Arends 2012	Cochrane Depression, Anxiety and Neurosis Review Group’s Specialised Register (CCDANCTR), up to October 2011. Cochrane Central Register of Controlled Trials (CENTRAL) up to Issue 4, 2011; MEDLINE, EMBASE, PsycINFO and ISI Web of Science, up to February 2011; WHO trials portal (ICTRP) and ClinicalTrials.gov in March 2011; RCTs	Acute or chronic adjustment disorders	Pharmacological interventions, psychological interventions, relaxation techniques, exercise programs, employee assistance programs or combinations of these interventions.	RTW (partial and full)	Based on 9 studies reporting on 10 psychological interventions and 1 combined intervention. Moderate evidence: cognitive behavioural therapy (CBT) did not significantly reduce time until partial RTW. Low evidence: CBT did not significantly reduce time to full RTW compared with no treatment. Moderate evidence: problem solving therapy significantly enhanced partial RTW at 1-year follow-up compared to non-guideline based care (MD -17.00, 95% CI -26.48 to -7.52) but did not significantly enhance time to full RTW at 1-year follow-up.
Cornelius 2011 [29]	January 1990-March 2009; PubMed, PsycINFO, EMBASE, CINAHL, Business Source Premier; English, German, French, Dutch; observational studies (i.e., case-control, cohort, longitudinal)	Mental disorders	Factors: nature and severity of mental disorder focusing on depression, anxiety disorder and substance use disorder; demographics; health service use; adequacy of treatment; coping strategies and social support	RTW, long-term disability	Based on 7 studies (4 cohorts). Strong evidence: older age (>50 years) is associated with continuing disability and longer time to RTW. Limited evidence: for the association of other personal factors (gender, education, history of previous sickness absence, negative recovery expectation, socioeconomic status), health-related (stress-related and shoulder/back pain, depression/anxiety disorder) and external i.e., job-related factors (unemployment, quality and continuity of occupational care, supervisor behavior) with disability and RTW. Long-term disability is mostly related to non-medical conditions.
Hensing 2004	Up to October 2002; MEDLINE, PsycINFO, SSCI; English, Danish, Norwegian, Swedish; no restrictions on study design	Pychiatric disorders	Factors: demographic, work-related, family and social network, psychosocial related to childhood and adolescence	Sickness absence, disability pension	Based on 28 studies (6 cross-sectional, 20 longitudinal/prospective, 2 register studies). Limited evidence: women have a higher frequency and incidence of sickness absence. Conflicting evidence: effect of gender on the duration of sickness absence; age; work-related factors, factors related to family and social networks, psychosocial factors; whether individuals were at greater risk for sickness absence and disability pension; alcohol problems associated with increased risk of sickness absence and disability pension.
Lagerveld 2010	1995-2008; PsycINFO, PubMed, Scopus; English; no study design restriction	Depression	Disorder-related factors (most commonly addressed); personal and work-related factors (less frequently addressed)	Work participation, work functioning	Based on 30 studies (half cross-sectional, half longitudinal). Work participation: strong evidence for the association between a long duration of the depressive episode and work disability. Moderate evidence for the associations between more severe types of depressive disorder, presence of co-morbid mental or physical disorders, older age, a history of previous sick leave, and work disability. Work functioning: moderate evidence that severe depressive symptoms were associated with work limitations and clinical improvement was related to work productivity.
Nieuwenhuijsen 2008	Up to August 2006; Cochrane Library CENTRAL register, MEDLINE, EMBASE, CINAHL, PsycINFO, OSH-ROM, NHS-EED; database of Abstracts of Reviews of Effectiveness; no language restrictions; RCTs	Depression	Work-directed (e.g., modified working hours and job tasks) and worker-directed interventions (e.g., pharmacological or psychological) aimed at reducing work disability.	Sickness absence	Based on 11 studies (worker-directed interventions). No evidence of an effect of medication alone, enhanced primary care, psychological interventions or the combination of those with medication on sickness absence of depressed workers.
Stergiopoulos 2011 [52]	Up to June 2011; MEDLINE, PsycINFO, EMBASE, Web of Science; English, French; no restrictions on study design	Work-related post-traumatic stress disorder	Work-related interventions	Work outcomes	Based on 7 articles (3 RCTs, 3 pre-post, 1 systematic review). Strong evidence that psychotherapy-based workplace interventions may be effective at improving work outcomes.
Brain Injury					
Baldwin 2011	Up to September 2009. CINAHL, AMED, MEDLINE, PsycINFO, Proquest 5000; English	Stroke survivorship	Vocational rehabilitation programs	RTW rates	Based on 6 studies (retrospective single cohort designs). Limited evidence: RTW rates ranged from 12% to 49%.
Fadyl 2009 [55]	1990-2007; MEDLINE, PsycINFO, CINAHL, AMED, Health and Psychosocial Instruments, Evidence-Based Medicine databases, Web of Science; English; no limit on study design	Traumatic brain injury	Vocational rehabilitation: (1) program-based vocational rehabilitation model, (2) supported employment model, (3) case coordination model	Employment outcomes	Based on 20 quantitative studies. (1) Program-based: weak evidence for better vocational outcomes (e.g., employment, wages, remain employed at 1 year following placement); (2) supported employment: weak evidence for gaining employment that lasted at least 90 days; (3) case coordination: moderate evidence for higher employment and productivity outcomes. Weak evidence that people who receive this intervention within the first year following injury are placed into employment more quickly No clear evidence to suggest what should be considered the “best practice” approach to vocational rehabilitation.
Nightingale 2007 [48]	Up to June 2006; MEDLINE, PsycINFO, EMBASE, CINAHL; English; cohort studies	Traumatic brain injury	Preinjury, injury, and early postinjury factors	RTW	Based on 27 studies. Limited evidence for preinjury employment, injury severity, cognitive factors, neurophysical factors, and multidimensional/participation factors.
Turner-Stokes 2005 [45]	Up to April 2008; CENTRAL (The Cochrane Library 2008, Issue 2), MEDLINE, EMBASE, ISI Web of Science: Science Citation Index Expanded (SCI-EXPANDED), ISIWeb of Science: Conference Proceedings Citation Index-Science (CPCI-S), Internet-based trials registers: ClinicalTrials.gov, Current Controlled Trials, and RehabTrials.org.; RCTs, quasi-randomized and quasi-experimental designs	Acquired brain injury	Multidisciplinary rehabilitation	RTW	Based on 2 RCTs regarding traumatic brain injury. Moderate-strong evidence for no significant differences between intervention and controls (appropriate information and advice).
van Velzen 2009	1992-July 2008; PubMed, EMBASE, CINAHL, PsycINFO; English, Dutch, German; no restrictions on study design	Acquired brain injury	Varying prognostic factors	RTW	Based on 22 studies. Strong evidence for no association or a negative association with RTW: gender, anatomic location, injury severity, depression, anxiety, inpatient length of stay. Weak evidence for trainable/treatable factors: ability to perform activities of daily living, residual physical deficits/higher disability level, number of associated injuries.
Willemse-van Son 2007	1995-April 2005; PubMed, PsycINFO; English, French, German, Dutch; prospective cohort studies	Traumatic brain injury	Varying prognostic factors	Activity limitations, participation restrictions	Based on 25 articles reporting on 14 cohorts. Strong evidence for predicting disability: older age, pre-injury unemployment, pre-injury substance abuse, and more disability at rehabilitation discharge. Strong prognostic factors for being non-productive: pre-injury unemployment, longer post-traumatic amnesia, more disability at rehabilitation admission, and pre-injury substance abuse.
Other					
Allebeck 2004	Up to October 2002; MEDLINE, PsycINFO, SSCI; English, Danish, Norwegian, Swedish; no restriction on study design	Any diagnosis or underlying disease	Varying prognostic factors	Sick leave, disability pension	Based on 96 studies (44 cross-sectional, 32 longitudinal, 7 cohort, 6 time series, 5 quasi-experimental, 2RCT). Family factors: no evidence that marital status or children living at home are associated with sickness absence; limited evidence for an effect of divorce. Work-related factors: limited evidence for an effect of physically stressful work; moderate evidence for low psychological control over the work situation. Limited evidence for a correlation in time between unemployment and sickness absence. Moderate evidence that the amount of sickness absence is influenced by the design of the social insurance system, but insufficient evidence on the magnitude of change required to influence the level of sickness absence. The same results apply to disability pension. Moderate evidence for the effects of socio-economic status.
de Boer 2011 [24]	Up to February 2010; Cochrane Central Register of Controlled Trials (CENTRAL, in *The Cochrane Library* Issue 2, 2010), MEDLINE, EMBASE, CINAHL, OSH-ROM, PsycINFO, DARE; RCTs, controlled before-after studies	Cancer	RTW interventions (e.g., psychological, vocational, physical, medical or multidisciplinary)	RTW	Based on 14 articles reporting 14 RCTs and 4 controlled before-after studies. Low evidence of similar RTW rates for psychological interventions compared to care as usual (OR 2.32, 95% CI 0.94- 5.71). No vocational interventions were retrieved. Very low evidence: physical training is not more effective than care as usual (OR 1.20, 95% CI 0.32- 4.54). Low quality evidence: functioning conserving approaches had similar RTW rates as more radical treatments (OR 1.53, 95% CI 0.95- 2.45). Moderate evidence: multidisciplinary interventions involving physical, psychological and vocational components led to higher RTW rates than care as usual (OR 1.87, 95% CI 1.07-3.27).
Dekkers-Sanchez 2008	Up to July 2007; MEDLINE, EMBASE, PsycINFO, Web of Science; no specified language restriction; cohort studies	Workers on sick leave for at least 6 weeks	Factors: predisposing, precipitating, perpetuating, individual or work-related	Long-term sick leave	Based on 5 cohort studies. Weak evidence that older age and history of sickness absence are associated with long-term sick leave. Insufficient evidence regarding individual or work-related factors. No evidence regarding perpetuating factors.
Detaille 2009 [22]	1990-2008; MEDLINE, EMBASE; English	Rheumatoid arthritis, chronic obstructive pulmonary disease, asthma, diabetes mellitus, or ischemic heart disease	5 groups of prognostic factors based on ICF: disease-related factors, body function or structural impairment factors, activity limitation and participation restriction factors, environmental factors, and personal factors	Work disability	Based on 43 cohort studies (20 rheumatoid arthritis, 3 asthma, 20 ischemic heart disease). Moderate-strong evidence that employees are at higher risk of work disability if they have: (i) a more severe chronic disease (disease-related factors), including a high level of perceived health complaints, (ii) disease-specific impaired body functions, such as pain and swollen/deformed joints in rheumatoid arthritis, depression in ischemic heart disease, sickness absence (body function or structural impairment factors) and (iii) more daily physical limitations caused by the disease (activity limitation and participation restriction factors). Other factors contributing to work disability are older workers (personal), women (personal), manual/blue-collar workers (environmental) and low-educated workers (personal).
Khan 2009	Cochrane Multiple Sclerosis Group’s Trials Register (February 2011), PEDro (1990-2011), ISI Science Citation Index (1981-2011), Cochrane Rehabilitation and Related Therapies Field trials Register, National Health Service National Research Register; no language restrictions; RCTs, controlled clinical trials	Multiple sclerosis	Vocational rehabilitation interventions	RTW and employment	Based on 2 trials (1 RCT, 1 clinical controlled). Insufficient evidence to support vocational rehabilitation interventions.
O’Neil 2010 [28]	1994-July 2009; PubMed, OVID, MEDLINE, PROQUEST, CINAHL plus, CCOHS, SCOPUS, Web of Knowledge; English; prospective cohort studies	Cardiac event (myocardial infarction, acute coronary syndrome, coronary artery disease)	Depression	Work resumption	Based on 12 articles (11 prospective cohort studies, 1 RCT). Strong evidence. Depression recorded between admission and up to 2 months post-discharge can significantly predict poorer RTW outcomes 6-12 months after a cardiac event. Other common predictors were age and patient perceptions of their illness and work performance.
Perk 2004	Up to October 2002; MEDLINE, PsycINFO, SSCI; English, Danish, Norwegian, Swedish; no restriction on study design	Stroke, coronary artery disease	Factors, interventions	Sick leave	Based on 33 cohort studies, 10 RCTs, 1 randomized trial, 1 case-control. Limited evidence: stroke: higher rate for younger patients RTW during first year post-stroke. Myocardial infarction: RTW is more rapid with percutaneous coronary intervention vs. coronary artery bypass grafting; no differences in long-term sick leave. People at higher ages or with physically demanding jobs RTW to a lesser degree.
Shepherd 2012	January 1999-November 2010; CINAHL, MEDLINE, PsycINFO; English; RCTs	Coronary heart disease	Cardiac rehabilitation interventions (publicly funded)	RTW	Based on 1 RCT. Limited evidence observed for earlier RTW.
Tamminga 2010 [53]	Up to October 2008; MEDLINE, PsycINFO, EMBASE, CINAHL; no language or study design restriction	Cancer	Work-directed interventions	RTW, employment status, work retention	Based on 7 studies (1 RCT, 3 controlled trials, 3 prospective cohort studies). The most frequently reported work-directed components were occupational training, encouragement, work advice, work accommodations, or education. Limited evidence that the intervention increased RTW.

*ICF* International Classification of Functioning, Disability, and Health, *LBP* low back pain, *MSK* musculoskeletal, *RTW* return to work

### Data synthesis

We determined which prognostic factors were common across health conditions (Table 2) based on the study authors’ conclusions of the accepted systematic reviews (Table 1). Factors were considered common if supported by consistent evidence from more than one health condition or injury; otherwise the results were considered inconclusive. We considered the evidence consistent if the findings in the majority of the systematic reviews in a category were in the same direction (i.e., the factor either had a positive, negative or no association with RTW outcomes) (Table 2).

**Table 2:** Common RTW prognostic factors

Study	Health condition	Direction of association
Common for positive effect on RTW outcomes		
Personal factors		
Higher education and socioeconomic status		
Clay 2010	MSK	+
Steenstra 2005	MSK	None
Cornelius 2011	Mental	+
Detaille 2009	Other	+
Allebeck 2004	Other	+
Higher self-efficacy/optimistic perceptions and expectations		
Clay 2010	MSK	+
Iles 2008	MSK	+
Kuijer 2006	MSK	+
Cornelius 2011	Mental	+
O’Neil 2010	Cardiac	+
Body structure and function factors		
Lower severity of injury/illness		
Clay 2010	MSK	+
Kuijpers 2004	MSK	+/−
Lagerveld 2010	Mental	+
Nightingale 2007	Brain injury	+
van Velzen 2009	Brain injury	None
Detaille 2009	Other	+
Environmental factors		
Multidisciplinary interventions		
Dick 2011	MSK	+
Hoffman 2007	MSK	+
Norlund 2009	MSK	+
Ravenek 2010	MSK	+/−
Tveito 2004	MSK	None
van Geen 2007	MSK	+
van Middlekoop 2011	MSK	+
Williams 2007	MSK	+
Désiron 2011	MSK + Other	+
Hoefsmit 2012	MSK + Other	+
van Oostrom 2009	MSK + Other	+
Turner-Stokes 2005	Brain injury	None
de Boer 2011	Cancer	+
Occupational care/training		
Cornelius 2011	Mental	+
Tamminga 2010	Cancer	+
Educational interventions		
Brox 2008	MSK	+
Heymans 2005	MSK	+
Liddle 2007	MSK	+
Meijer 2005	MSK	+/−
Tveito 2004	MSK	None
Tamminga 2010	Cancer	+
Psychological interventions		
Meijer 2005	MSK	+/−
Palmer 2012	MSK	+
van Middlekoop 2011	MSK	+
Corbière 2006	MSK + Mental	+
Arends 2012	Mental	–
Nieuwenhuijsen 2008	Mental	Insufficient
de Boer 2011	Cancer	+
Stakeholder participation in RTW process		
Carroll 2010	MSK	+
Franche 2005	MSK	+
Cornelius 2011	Mental	+
Work modification/accommodation		
Carroll 2010	MSK	+
Dick 2011	MSK	+
Franche 2005	MSK	+
Palmer 2012	MSK	+
Williams 2007	MSK	+
Tamminga 2010	Cancer	+
RTW coordination		
Franche 2005	MSK	+
Schandelmaier 2012	MSK + Other	+
Early intervention		
Carroll 2010	MSK	+
Hoefsmit 2012	MSK + Other	+
Outpatient/comprehensive treatment		
Hlobil 2005	MSK	_+_
Tveito 2004	MSK	_+_
Shepherd 2012	Coronary heart disease	_+_
Activities and participation factors		
Preinjury employment		
Hansson 2004	MSK	+
Cornelius 2011	Mental	+
Nightingale 2007	Brain injury	+
Common for negative effect on RTW outcomes		
Personal factors		
Older age		
Hansson 2004	MSK	–
Steenstra 2005	MSK	–
Verkerk 2012	MSK	+/−
Cornelius 2011	Mental	–
Hensing 2004	Mental	+/−
Lagerveld 2010	Mental	–
Willemse-van Son 2007	Brain injury	–
O’Neil 2010	Cardiac	–
Perk 2004	Stroke, Coronary artery disease	–
Dekkers-Sanchez 2008	Other	–
Detaille 2009	Other	–
Female		
Hansson 2004	MSK	+/−
Steenstra 2005	MSK	–
Verkerk 2012	MSK	+/−
Cornelius 2011	Mental	–
Hensing 2004	Mental	–
van Velzen 2009	Brain injury	None
Detaille 2009	Other	–
Body structure and function factors		
Higher pain/impairment/disability		
Hansson 2004	MSK	–
Kent 2008	MSK	+/−
Kuijpers 2004	MSK	+/−
Steenstra 2005	MSK	–
Verkerk 2012	MSK	–
Cornelius 2011	Mental	–
Willemse-van Son 2007	Brain injury	–
Detaille 2009	Other	–
Depression		
Iles 2008	MSK	None
Cornelius 2011	Mental	–
Lagerveld 2010	Mental	–
Van Velzen 2009	Brain injury	None
O’Neil 2010	Cardiac	–
Detaille 2009	Other	–
Environmental factors		
Higher physical work demands		
Hansson 2004	MSK	–
Steenstra 2005	MSK	–
Verkerk 2012	MSK	–
Perk 2004	Stroke, Coronary artery disease	–
Allebeck 2004	Other	–
Activities and participation factors		
Previous sick leave		
Hansson 2004	MSK	–
Kuijpers 2004	MSK	+/−
Cornelius 2011	Mental	–
Lagerveld 2010	Mental	–
Willemse-van Son 2007	Brain injury	–
Allebeck 2004	Other	–
Dekkers-Sanchez 2008	Other	–
Detaille 2009	Other	–
Activity limitation/participation restriction		
Kent 2008	MSK	+/−
Verkerk 2012	MSK	+/−
Lagerveld 2010	Mental	–
Nightingale 2007	Brain injury	–
Van Velzen 2009	Brain injury	–
Detaille 2009	Other	–
Common for no effect on RTW outcomes		
Body structure and function factors		
Anxiety and stress		
Iles 2008	MSK	None
Cornelius 2011	Mental	–
Van Velzen 2009	Brain injury	None
Inconclusive		
Personal factors		
Marital status/Nnumber of dependents		
Steenstra 2005	MSK	None
Allebeck 2004	Other	None
Lower social support		
Steenstra 2005	MSK	–
Hensing 2004	Mental	+/−
Perceived high work demands		
Hansson 2004	MSK	–
Hensing 2004	Mental	Insufficient
Higher work satisfaction		
Hansson 2004	MSK	+
Iles 2008	MSK	None
Steenstra 2005	MSK	None
Dekkers-Sanchez 2008	Other	Insufficient
Smoking		
Hansson 2004	MSK	None
Steenstra 2005	MSK	None
Fear avoidance beliefs		
Iles 2008	MSK	–
Psychosocial factors		
Kent 2008	MSK	+/−
Hensing 2004	Mental	+/−
Medical history/co-morbidities		
Kent 2008	MSK	+/−
Steenstra 2005	MSK	None
Lagerveld 2010	Mental	_
Alcohol problems		
Hensing 2004	Mental	+/−
Willemse-van Son 2007	Brain injury	–
Body structure and function factors		
Clinical factors		
Kent 2008	MSK	+/−
van Velzen 2009	Brain injury	None
Environmental factors		
Workplace social support		
Campbell 2013	MSK	+
Hansson 2004	MSK	None
Hensing 2004	Mental	Insufficent
Higher compensation		
Clay 2010	MSK	–
Iles 2008	MSK	Insufficient
Steenstra 2005	MSK	–
Working longer shifts		
Steenstra 2005	MSK	None
Occupation type/White vs. blue collar		
Clay 2010	MSK	None
Kuijpers 2004	MSK	+/−
Steenstra 2005	MSK	None
Hensing 2004	Mental	Insufficient
Detaille 2009	Other	+
Company size		
Steenstra 2005	MSK	None
Higher locus of control/decision latitude		
Iles 2008	MSK	Insufficient
Hoefsmit 2012	MSK + Other	+/−
Hensing 2004	Mental	Insufficient
Allebeck 2004	Other	+
Encouragement		
Tamminga 2010	Cancer	+/−
Exercise		
Oesch 2010	MSK	+
Palmer 2012	MSK	+
Tveito 2004	MSK	+
Pamphlet		
Tveito 2004	MSK	None
Enhanced primary care/Medication		
Nieuwenhuijsen 2008	Mental	Insufficient
Early contact with worker by workplace		
Franche 2005	MSK	+
Williams 2007	MSK	+
Graded activity/exposure		
Macedo 2010	MSK	+/−
van der Giessen 2012	MSK	+/−
Physical and work conditioning		
Meijer 2005	MSK	+/−
Schaafsma 2013	MSK	+
Back belts		
Tveito 2004	MSK	None
Lower-intensity intervention (vs. higher)		
Palmer 2012	MSK	+
van Geen 2007	MSK	None
Hoefsmit 2012	MSK + Other	+/−
de Boer 2011	Cancer	none
Activation & time-contingent interventions		
Hoefsmit 2012	MSK + Other	+/−
Disability management programs/Generic interventions		
Gensby 2012	MSK + Other	Insufficient
Hoefsmit 2012	MSK + Other	+/−
Psychotherapy-based workplace interventions		
Stergiopoulos 2011	Mental	+
Vocational rehabilitation programs		
Baldwin 2011	Brain injury	+
Fadyl 2009	Brain injury	+
Khan 2009	Multiple sclerosis	Insufficient

Direction of association with RTW: positive (+), negative (−), conflicting (+/−), no association (none), or insufficient evidence

*MSK* musculoskeletal, *RTW* return to work

We organized the evidence using the domains of the International Classification of Functioning, Disability and Health (ICF) framework [19]. The ICF is a model of functioning and disability with a biopsychosocial approach [20, 21], and has been previously used to examine RTW factors across different health conditions [22]. The ICF classification is structured into four broad components or domains [19, 21]: (1) Personal (e.g., age, sex); (2) Body Structure and Function (e.g., disease/injury-related factors); (3) Environmental (e.g., all factors related to work conditions, work environment, work support and accommodation, etc.); and (4) Activity Limitations and Participation Restriction (e.g., history of sickness absence, inability to perform some activities of daily living).

## Results

**Fig. 1:**
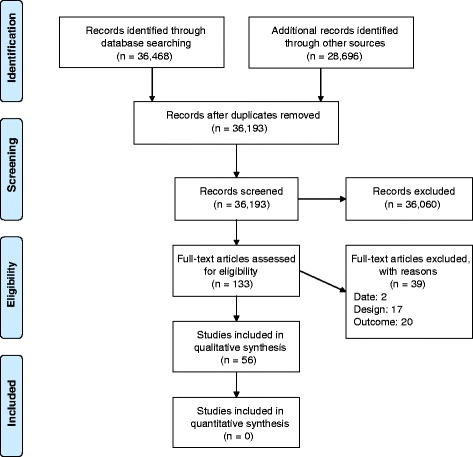
Flow chart of literature search

A total of 36,468 records were identified in the electronic database search and 28,696 records were identified from the grey literature (Fig. 1). After duplicates were removed 36,193 titles remained. After applying the inclusion and exclusion criteria to titles and abstracts, 36,060 records were excluded and 133 full-text articles were assessed for eligibility. Of these, 94 articles were eligible and critically reviewed and 38 were deemed inadmissible due to having a high risk of bias. We deemed the majority of these reviews inadmissible primarily because they did not assess the quality of primary studies and synthesize the results according to study quality. Thus, our findings are based on the synthesis of 56 systematic reviews.

### Study characteristics

Over half of the studies included MSKDs alone (*n* = 29) or in combination with other conditions (*n* = 6); and the majority examined spine-related disorders including neck and LBP (*n* = 26) (Table 1). Studies also included mental health disorders (*n* = 9), traumatic and acquired brain injury (*n* = 6), cardiovascular conditions (*n* = 4), cancer (*n* = 2), stroke (*n* = 1), multiple sclerosis (*n* = 1), other chronic illness (*n* = 1), as well as any underlying disease (*n* = 1). The search dates of the systematic reviews spanned database inception to 2012. Most of the primary studies were conducted in workplace or clinical settings. The majority of reviews explored interventions (*n* = 38) while others explored non-intervention factors (*n* = 19) (i.e., personal, disease-specific, environmental, or activity-related factors). A summary of the common RTW prognostic factors across health conditions is in Table 2.

### Common prognostic factors associated with positive RTW outcomes

Personal, body structure and function, as well as activity-related factors associated with positive RTW outcomes included higher education and socioeconomic status, higher self-efficacy/optimistic perceptions and expectations, lower severity of the injury/illness, and being employed preinjury. Environmental factors associated with positive RTW outcomes included stakeholder participation in the RTW process, work modification/accommodation, and RTW coordination. Many interventions, especially those involving a workplace component, were associated with positive RTW outcomes, including multidisciplinary, occupational care/training, education, psychological, and outpatient interventions/comprehensive treatment. Additionally, early intervention, initiated within the first 6 weeks, was linked to positive RTW outcomes. Stakeholder participation included communication among stakeholders, including between the supervisor and employee, worker and the workplace, and the healthcare provider and the workplace, as well as a meeting bringing all stakeholders together. Types of accommodations included lighter and modified duties, and adjusting work schedules, tasks and the physical worksite. Multidisciplinary interventions involved multiple resources including professionals from more than one discipline (e.g., occupational health physician, case-coordinator, physical therapist and others) who deliver a variety of intervention elements (e.g., exercise, education, behavioral treatment, vocational advice, etc.) with or without the inclusion of other stakeholders (e.g., supervisors, employers, insurance representatives). Examples of multidisciplinary intervention element combinations included, physical, educational, psychological and social [23], as well as physical, psychological and vocational interventions [24]. Educational interventions included back schools, fear-avoidance training, and work advice. Psychological interventions included cognitive-behavioural therapy and problem solving therapy. Comprehensive treatment refers to focusing on several of the factors known to be associated with a health condition, for example, psychological factors in addition to physical factors in LBP [25].

### Common prognostic factors associated with negative RTW outcomes

Factors from all ICF domains were associated with negative RTW outcomes. These included older age, being female, higher pain or disability, depression, previous sick leave, activity limitations/participation restriction (e.g., limited ability to perform activities of daily living (ADLs) and periods of unemployment), and higher physical work demands.

### Common prognostic factors having no association with RTW outcomes

The only factor we found that was not associated with RTW outcomes was having anxiety or stress, in the body structure and function domain.

### Prognostic factors with inconclusive evidence regarding RTW outcomes across health conditions

We could not come to any firm conclusions regarding many prognostic factors. A number of factors have not been studied across different health conditions including fear avoidance beliefs, intensity of the intervention (e.g., low vs. high), and work conditioning. Most of these factors have been studied in MSKD studies alone. For instance, common factors that were associated with positive RTW outcomes in MSKD studies included interventions that included exercise and early contact with the worker by the workplace (i.e., within the first three months following onset of work disability) [26]. Also in MSKD studies, receiving higher compensation (e.g., higher weekly wage compensation rates from workers’ compensation due to occupational back injuries) [27] was commonly associated with negative RTW outcomes; smoking and level of work satisfaction showed no association with RTW outcomes. Findings were conflicting with respect to a number of other factors such as type of occupation, and vocational rehabilitation programs.

## Discussion

### Summary of findings

To our knowledge this is the first systematic review of systematic reviews that assessed RTW outcomes across health conditions and injuries. We critically reviewed 94 systematic reviews and conducted a best evidence synthesis on 56 reviews with a low risk of bias relating to RTW; over half of these addressed MSKDs. The other half explored mental health disorders, brain injury, cardiovascular conditions, cancer, stroke, and multiple sclerosis. While our search included all conditions, only few have actually been studied. Many factors have been assessed, but only a few were common across conditions. Where factors have been reviewed across conditions, the results are generally in the same direction for a number of factors, suggesting that other common factors may exist across conditions. RTW outcomes were influenced by prognostic factors in all four ICF domains. Common factors associated with positive RTW outcomes were higher education and socioeconomic status, higher self-efficacy and optimistic expectations for recovery and RTW, lower severity of the injury/illness, RTW coordination, and multidisciplinary interventions that include the workplace and stakeholders. Common factors associated with negative RTW outcomes were older age, being female, higher pain or disability, depression, higher physical work demands, previous sick leave and unemployment, and activity limitations. Factors related to the specific illness or injury did not impact RTW outcomes. In other words, in many cases, it is likely that the health condition itself is not that important in influencing RTW. Our findings confirm those of Briand et al. [14], that prognostic factors other than disease-specific factors are associated with RTW outcomes. Our results also align, in that the important components of RTW interventions are RTW coordination, occupational training or conditioning, workplace-based interventions, work accommodations, and contact between the various stakeholders. A major finding our review adds is that these factors are relevant for other conditions, not just MSKDs.

### “New” modifiable prognostic factors

Identifying modifiable prognostic factors is of utmost importance because these could respond to new interventions targeted at modifying them. We found that expectations of recovery and RTW, pain and disability levels, depression, workplace factors, and access to multidisciplinary resources are important modifiable factors in progressing RTW across health and injury conditions.

Having optimistic expectations for recovery and RTW was commonly associated with positive RTW outcomes, and these findings are represented by evidence from studies on myocardial infarction [28] as well as mental health [29] and MSKDs [30, 31]. This factor is also potentially modifiable [32–35]. Those expecting to recover more slowly after injury often do [36–38], and not expecting to RTW leads to a slower recovery [39] and a higher risk of receiving sick leave benefits [40]. This suggests the importance of identifying RTW expectations early on. Negative RTW expectations were also associated with longer time to RTW across MSK and mental health disorders and other physical injuries [34]. Thus, regardless of the health condition or injury, asking whether a worker expects to recover and RTW, especially early on, can help identify those at high risk for delayed RTW. Clinicians should also be trained to better understand this process and not, inadvertently, contribute to negative RTW expectations. For example, a recent study found that a significant proportion of clinicians believed that people with a psychotic disorder are not capable of any kind of work [41]. Thus, stigma and discrimination in mental health conditions may have an impact on expectations of RTW, and on RTW outcomes.

The level of pain, impairment, or disability and one’s experiences is often multi-factorial and not directly or completely attributable to disease-specific factors, especially in the long term [42]. For example, in individuals with mild traumatic brain injury, more severe injuries were associated with a higher level of physical and cognitive symptoms at 3 months, but not at later follow-ups [43]. Conceivably, general interventions targeting one’s ability to cope with pain or disability early on, regardless of the contributing disease-specific factor, may ultimately help to improve RTW outcomes. Likewise, identifying and managing depression (regardless of the initial source of depression) in ill or injured workers, irrespective of the traceable disease-specific factor, may additionally lead to improved RTW outcomes.

Multidisciplinary RTW interventions, especially occurring at the workplace, are supported from studies of cancer [24], MSKDs [14] and mental health disorders [44]. Our findings suggest it is important to at least provide access to multiple resources including health and occupational professionals who can deliver a combination of interventions when and to whom it is required; of particular importance is ensuring these resources are available for conditions with a less favourable prognosis. For example, interventions beyond information and advice are not required to improve RTW outcomes in those with mild traumatic brain injury [45]. In contrast, more complex interventions involving physical, vocational and psychological elements do improve RTW outcomes in patients with cancer [24]. Similarly, multidisciplinary RTW coordination programs improve outcomes across more chronic MSKDs [46]. Thus, providing access to multidisciplinary resources may better address the multi-factorial nature of RTW [29, 47–49] and help improve outcomes across complex conditions such as chronic LBP, which contributes enormously to the burden of work disability [1, 50]. Clinicians should, however, remain mindful that too much health care too early after an injury (e.g., mild traumatic brain injury, whiplash) can delay recovery [51].

Work accommodation is an important factor for improved RTW outcomes across health and injury conditions. The availability of different levels of work accommodation are supported by systematic reviews on mental health [52], MSKDs [26], cancer [53], as well as other chronic illnesses and disability [26]. Work accommodation can include for example offering lighter or modified duties for those who suffered a work-related MSK injury (e.g., acute low back pain), as well as offering graded work exposure or an onsite work evaluation for those with work-related post-traumatic stress disorder [52]. We also found that the presence of a RTW plan and/or case-coordinator was important. Developing a RTW plan and/or having a case-coordinator in place to implement this plan, helped improve RTW outcomes for employees with general disability [46], MSKDs [54] and brain injuries [48, 55]. Similarly, for MSKDs, Briand et al. [14] found that centralizing the management of the RTW process by way of a multidisciplinary team working in collaboration with the workplace can improve RTW outcomes. Within these teams, there is access to multiple resources that can assess the multiple causes of work disability as well as implement specific interventions as required. Applying this same centralized team approach may help improve RTW outcomes in other complex non-MSKD conditions as well as foster collaboration with the workplace. In turn, this may also help improve stakeholder awareness [56] as well as interpersonal communication. We found interpersonal communication involving early contact and with multiple stakeholders to be another common prognostic factor associated with positive RTW outcomes.

Other than older age and being female, the majority of negative RTW factors we found are also modifiable. These include having higher physical work demands, previous sick leave, or activity limitations/participation restriction. Taken together, the modifiable factors discussed here could be extended further to other conditions and likely help inform better RTW processes.

### Comparison with the “seven principles of successful RTW”

Our findings support the “seven principles for successful RTW” previously established for MSKDs [13] for the most part. We did not come across any studies related to two of the principles - supporting the returning worker without disadvantaging co-workers and supervisors; and having supervisors trained in work disability prevention. Our literature search did not include any systematic reviews prior to 2004 and did not include any qualitative studies. Nonetheless, it is commonsensical to want to avoid disadvantaging others while supporting the returning worker. Further, it is reasonable for supervisors to receive some work disability prevention training to try to improve RTW outcomes.

### Strengths and limitations

Our review has several strengths including comprehensive search strategies and an in-depth methodological quality assessment of individual systematic reviews. Our review also has limitations. First, only one reviewer screened the titles and abstracts. However, citations were only deemed irrelevant if the title or abstract did not include any information on RTW outcomes. Therefore, the potential for excluding relevant studies was low. Second, we did not assess the risk of bias for the primary studies cited in the systematic reviews we accepted; thus we cannot be certain of their quality. We based our findings on the authors’ conclusions of the systematic reviews. Third, the majority of reviewed studies were based on MSKDs. As a result, some of these systematic reviews may have reviewed the same studies and even interpreted the quality of the evidence differently. Other limitations include possible publication bias and the potential for missing relevant reviews and/or primary studies not captured in the systematic reviews we included in this paper. Despite these potential limitations, we believe our findings are robust enough to help inform both RTW strategies across health conditions and injuries and future research efforts.

### Clinical implications

Primary studies identifying more non-modifiable prognostic factors (e.g., age, sex, and specific disease-related factors), especially in MSKDs, offer little added value in helping to improve RTW outcomes and address the burden of work disability. Work-related factors (e.g., RTW coordination, work accommodations), depression, pain and disability, as well as certain psychosocial factors (e.g., expectations of recovery and RTW) are important RTW predictors and some of these can already be modified with specific interventions. Modifiable factors may be influenced by policy and practices which may vary between countries. By targeting modifiable factors with this in mind, RTW outcomes may be improved. Given our findings, we support an expanded set of common RTW principles across health conditions for use by employers, health care providers and other stakeholders (Table 3). This set includes the seven original principles by the IWH, and an additional principle given our findings - the worker has access to multidisciplinary resources (including clinical interventions for the management of pain, disability, depression and poor expectations for recovery), where necessary, working in combination with the other stakeholders. We emphasize that while providing multidisciplinary resources in concert with the workplace is important, clinicians need to be educated about the risk of iatrogenic disability [51] and take steps to prevent this. For example, workers should only remain off work if it is medically necessary [57], and clinicians should refrain from giving carte blanche permission for their patients to remain off work indefinitely to receive ongoing therapy of marginal value. Overall, multiple countries endorse similar recommendations for injured/ill workers but these are most distinctly expressed by the IWH in Canada through their seven principles of successful RTW. For this reason, we chose to relate our findings to these seven principles and incorporate our results.

**Table 3:** Common principles for successful return to work

Common principles for successful return	
1. The workplace has a strong commitment to health and safety, which is demonstrated by the behaviours of the workplace parties.^a^ 2. The employer makes an offer of modified work (also known as work accommodation) to injured/ill workers so they can return early and safely to work activities suitable to their abilities.^a^ 3. RTW planners ensure that the plan supports the returning worker without disadvantaging co-workers and supervisors.^a^ 4. Supervisors are trained in work disability prevention and included in RTW planning.^a^ 5. The employer makes early and considerate contact with injured/ill workers.^a^ 6. Someone has the responsibility to coordinate RTW.^a^ 7. Employers and health-care providers communicate with each other about the workplace demands as needed, and with the worker’s consent.^a^ 8. The worker has access to multidisciplinary resources (including clinical interventions for the management of pain, disability, depression and poor expectations for recovery), where necessary, working in combination with the other stakeholders.	

*RTW* return to work

^a^ The first seven principles are the original *Seven Principles for Successful Return to Work* by the Institute for Work and Health (IWH)^9^

### Future direction for research

Psychosocial and pain- and work-related factors can be tested together in clinical trials across a variety of health and injury conditions. Studies also need to identify which factors (e.g., health history, cultural, work and family influences, pain beliefs, etc. [58]) influence recovery and RTW expectations and might be modified with specific interventions. A previous consensus panel of expert opinion found that expectations of recovery are likely modifiable, and as these have a high impact on RTW should be a priority for future research [59]. Large prospective cohort studies would be helpful in detecting prognostic factors over longer periods of time, such as in the Whitehall studies [60], where evidence of differentials in socioeconomic status, earnings, and decision latitude impacting on work outcomes has emerged strongly. Qualitative or mixed methods studies may offer insight into the mechanisms that may explain how modifiable factors operate and contextual variations. Since RTW coordinators appear important to improving RTW outcomes; core competencies established for these individuals can be applied broadly to help improve RTW outcomes [61]. Finally, inconclusive and conflicting results are likely due in part to the heterogeneity of the study populations, varying measurement of the outcomes, and other methodological variations. Therefore, more high-quality evidence is still needed regarding prognostic factors for which the findings are still inconclusive, and to identify modifiable RTW prognostic factors across other, non-MSKDs.

## Conclusions

We synthesized the evidence from 56 systematic reviews regarding common prognostic factors that influence RTW outcomes across health conditions. This review establishes the importance of modifying workplace factors, pain, disability, depression, worker expectations as well as providing access to multidisciplinary resources in promoting positive RTW outcomes across different health and injury conditions. Employers, healthcare providers, and other stakeholders may use as a guide our updated Common Principles for Successful RTW, an expansion of the principles produced by the IWH, to facilitate RTW for injured/ill workers.

## Abbreviations

MSKD, musculoskeletal disorders; RTW, return to work
